# Analysis and comparison of the trends in burden of low back pain in China and worldwide from 1990 to 2021

**DOI:** 10.1186/s41043-025-00768-8

**Published:** 2025-02-13

**Authors:** Yongcun Wei, Yanchun Xie, Anwu Xuan, Hongwen Gu, Yi Lian, Zening Wang, Hongwei Wang, Hailong Yu

**Affiliations:** 1 Department of Orthopedics, General Hospital of Northern Theater Command of Chinese PLA, Shenyang, Liaoning 110016 China; 2 Liaoning University of Traditional Chinese Medicine Graduate School, Shenyang, Liaoning 110847 China

**Keywords:** Low back pain, Trend, Incidence, Prevalence, Disability-adjusted life years

## Abstract

**Background:**

Low back pain (LBP) substantially affects quality of life and functional capacity, ranking as a major global cause of disability. While the global burden of LBP has been extensively studied, China’s unique demographic, socioeconomic, and healthcare contexts warrant focused attention. As the world’s most populous nation undergoing rapid urbanization and aging, China presents a distinct landscape for LBP epidemiology. This study aims to chart the temporal shifts in the age- and sex-specific burdens of LBP in China from 1990 to 2021, encompassing incidence, prevalence, and disability-adjusted life years (DALYs). By benchmarking these trends against the worldwide disease burden, this research provides critical insights into how China’s experience aligns with or diverges from global patterns, offering valuable guidance for targeted public health strategies.

**Methods:**

This study leveraged open-access data from the Global Burden of Disease (GBD) repository, spanning the years 1990 to 2021, to scrutinize the epidemiological profile of LBP in China and across the globe. The analysis encompassed fluctuations in LBP incidence, prevalence, and DALYs. The Joinpoint regression model was employed to determine the average annual percentage change (AAPC) and its associated 95% confidence interval (95% CI), thereby quantifying the trajectory of LBP burden. A multifaceted comparative evaluation was performed to elucidate disparities in LBP burden between China and other regions, examining various aspects such as age, gender, and temporal dynamics.

**Results:**

From 1990 to 2021, both China and the world experienced a decline in age-standardized metrics related to LBP. In China, the age-standardized incidence rate (ASIR) decreased from 2,859.383 to 2,342.459 per 100,000, while globally, it fell from 3,534.988 to 3,176.63 per 100,000. Similarly, the age-standardized prevalence rate (ASPR) in China declined from 6,635.488 to 5,342.1 per 100,000, compared to a global reduction from 8,391.582 to 7,463.13 per 100,000. The age-standardized DALYs rate (ASDR) in China also dropped from 749.026 to 603.033 per 100,000, while globally, it decreased from 937.339 to 832.179 per 100,000. Notably, according to the AAPC results, China showed a more pronounced decrease in these metrics compared to the global averages, especially before 2015. Gender differences were evident, with women consistently exhibiting higher incidence, prevalence, and DALYs for LBP across all age groups and years. Age-related disparities were also significant: in 2021, the crude incidence rate (CIR), crude prevalence rate (CPR), and crude DALY rate (CDR) peaked in the 85–89 age group, reflecting the substantial burden of LBP among older adults. However, the highest number of incidence, prevalence, and DALYs was observed in the 55–59 age group, indicating a shift toward middle-aged individuals as a key affected population. Overall, while China’s LBP burden demonstrated a consistent decline, the gender and age patterns suggest a need for tailored public health interventions targeting middle-aged and elderly populations, as well as women who are disproportionately affected.

**Conclusion:**

Although China’s LBP burden has declined, it remains significant among middle-aged and elderly populations, with women disproportionately affected. Public health efforts should focus on ergonomic improvements, promoting physical activity, and accessible nonpharmacological treatments. Integrating LBP care into primary healthcare is vital to mitigate its impact and support the aging population.

**Supplementary Information:**

The online version contains supplementary material available at 10.1186/s41043-025-00768-8.

## Introduction

Low back pain (LBP) is a significant global health concern, affecting a substantial portion of the population and resulting in considerable disability and economic strain worldwide [1]. Globally, LBP is the leading cause of years lived with disability (YLDs), and its prevalence has been increasing over recent decades [2]. While advances in clinical practice guidelines (CPGs) have emphasized nonpharmacological interventions, such as exercise therapy, psychological support, and lifestyle adjustments, the global burden of LBP remains substantial [3–5]. An estimated 619 million individuals experienced LBP in 2020, a figure projected to rise to 843 million by 2050 due to factors such as aging populations, increased sedentary behavior, and work-related risks [6].

In the context of China, the burden of LBP has become an urgent public health issue due to the nation’s rapid urbanization and demographic shifts. Swift urban expansion has contributed to lifestyle changes, including prolonged sedentary behavior and occupational risks associated with desk-based jobs, which are major contributors to the rising prevalence of LBP [7, 8]. Despite this, much of the existing research on LBP has focused on global or regional trends, often neglecting the unique contexts of individual nations [9]. Studies from the Global Burden of Disease (GBD) initiative have explored the burden of LBP globally and regionally, highlighting its association with socioeconomic development. However, such studies often fail to capture the nuanced, country-specific trends that are essential for designing tailored public health interventions [10].

China, as the world’s most populous country, offers a unique and critical context for understanding the evolving burden of LBP. While several studies have investigated the incidence, prevalence, and YLDs associated with LBP in China, there is a notable lack of comprehensive, long-term analyses that assess its trends over time, especially regarding the influence of demographic and occupational shifts [11]. This gap underscores the need for a more granular understanding of LBP’s burden and its trajectory in China to inform public health policy and resource allocation.

To address this gap, the present study leverages the latest GBD data to conduct an in-depth analysis of the burden of LBP in China and its comparison to global trends from 1990 to 2021. By employing Joinpoint regression analysis, we examine the temporal dynamics of LBP and assess its evolution over three decades, disaggregated by age and gender. The findings aim to provide critical insights for policymakers, enabling evidence-based planning for preventive strategies and the effective allocation of public health resources [12, 13].

## Methods

### Data source

For this study, data were sourced from the GBD 2021 repository, which provides estimates for years lived with disability (YLDs), years of life lost (YLLs), disability-adjusted life-years (DALYs), and healthy life expectancy (HALE) across 371 diseases and injuries from 100,983 data sources [14]. These include vital records, population censuses, surveys, and health service data. The GBD database standardizes data to ensure comparability across time, geography, and demographics. LBP was defined as lasting at least one day annually, involving the lumbar spine, sacral segments, sacroiliac joints, and surrounding tissues such as muscles and ligaments [8, 15]. Variations in how LBP was defined across data sources posed challenges. To address these, the Bayesian meta-regression tool DisMod-MR was used to harmonize epidemiological parameters, adjust for differences in definitions, and minimize biases.

The study spans data from China and global trends over 1990–2021. Temporal and regional data gaps were managed using GBD’s predictive modeling and imputation techniques, supported by multiple data sources. Differences in data quality over time were addressed through age-standardized rates (ASR) to ensure consistency. Temporal variations in data were carefully managed by selecting datasets that adhered to standardized methodologies. By leveraging the extensive data aggregation and standardization processes of the GBD platform, this study provided reliable estimates for the trends and burden of LBP in China and globally. Despite inherent limitations in working with diverse datasets, the rigorous approach ensured meaningful insights into the temporal and geographical dynamics of LBP.

### Statistical analysis

Our analysis reviewed LBP metrics, including incidence, prevalence, DALYs, age-standardized incidence rate (ASIR), age-standardized prevalence rate (ASPR), and age-standardized DALY rate (ASDR) from the GBD database for China and globally. Additionally, we examined the crude incidence rate (CIR), crude prevalence rate (CPR), and crude DALY rate (CDR) for various age cohorts. To address potential confounders such as socio-economic status, healthcare infrastructure, and reporting quality differences, we relied on the GBD’s DisMod-MR modeling, which harmonizes data across sources to minimize bias. However, regional disparities may still contribute to residual confounding. Age-standardized rates were calculated based on the WHO world standard population used in GBD studies, ensuring comparability across regions and time. These metrics mitigate the effects of demographic differences, particularly relevant to China’s aging population.

Joinpoint regression analysis was employed to calculate the Annual Percentage Change (APC) and Average Annual Percent Change (AAPC) over the entire study period, along with their corresponding 95% confidence intervals (CI). The logarithmic transformation of age-standardized indicators was applied to a regression model: ln(y) = α + βx + ε, where y denotes the age-standardized indicator, and x signifies the calendar year. The AAPC was derived as 100 × (exp(β) − 1), with the 95% CI also being deduced from the model. An AAPC’s 95% CI exceeding 0 indicates an upward trend, less than 0 a downward trend, and inclusion of 0 a stable trend. The logarithmic transformation in our regression model was selected to linearize exponential trends, stabilize variance, and improve model fit. While alternative transformations (e.g., square root) were considered, the logarithmic approach offered the most consistent results with prior GBD studies and best practices in epidemiological research. Statistical processing and data visualization were executed with R statistical software (version 4.4.1) and the Joinpoint software (version 4.9.1.0), adopting a P value threshold of < 0.05 for statistical significance.

## Results

### The burden of LBP in China and globally

#### Incidence due to LBP in China and globally

From 1990 to 2021, the incidence of LBP in China escalated from 29,843,970 cases (95% CI: 26,065,824 − 34,012,369) to 43,374,995 cases (95% CI: 37,494,376 − 49,159,184), marking an aggregate augmentation of 45.339%. Globally, the number of LBP cases rose from 165,063,882 (95% CI: 145,785,270 − 185,933,884) in 1990 to 266,873,321 (95% CI: 235,369,489 − 299,406,380) in 2021, indicating a cumulative escalation of 61.679%. Regarding ASIR, globally, it declined from 3,534.988 per 100,000 individuals (95% CI: 3,133.037-3,960.989) in 1990 to 3,176.63 per 100,000 individuals (95% CI: 2,811.817-3,562.291) in 2021. In China, the ASIR fell from 2,859.383 per 100,000 individuals (95% CI: 2,508.615-3,225.529) in 1990 to 2,342.459 per 100,000 individuals (95% CI: 2,058.054-2,639.322) in 2021. Furthermore, China’s AAPC in the incidence rate dropped by 0.627% (95% CI: -0.671 to -0.583) from 1990 to 2021, while globally it reduced by 0.347% (95% CI: -0.371 to -0.322)(Table 1).

**Table 1:** The incidence, prevalence, and DALYs cases for LBP across all age groups, as well as the age-standardized rates, were analyzed for China and globally in the years 1990 and 2021. AAPC were also calculated

Location	Measure	1990					2021		1990-2021AAPC
		All-ages cases	Age- standardized rates per 100,000 people				All-ages cases	Age-standardized rates per 100,000 people	
		*n* (95% CI)	*n* (95% CI)				*n* (95% CI)	*n* (95% CI)	*n* (95% CI)
China	Incidence	29,843,970 (26,065,824 − 34,012,369)	2,859.383 (2,508.615-3,225.529)		43,374,995 (37,494,376 − 49,159,184)			2,342.459 (2,058.054-2,639.322)	-0.627 (-0.671 - -0.583)
	Prevalence	68,281,006 (59,158,022–77,853,897)	6,635.488 (5,770.679-7,472.799)		100,093,746 (87,128,173 − 113,014,316)			5,342.1 (4,660.413-5,976.282)	-0.682 (-0.728 - -0.636)
	DALYs	7,772,958 (5,520,145 − 10,545,676)	749.026 (530.011-1013.842)				11,297,805 (7,931,468 − 15,328,056)	603.033 (427.628–810.16)	-0.682 (-0.726 - -0.638)
Global	Incidence	165,063,882 (145,785,270 − 185,933,884)	3,534.988 (3,133.037-3,960.989)				266,873,321 (235,369,489 − 299,406,380)	3,176.63 (2,811.817-3,562.291)	-0.347 (-0.371 - -0.322)
	Prevalence	386,731,361 (341,581,662 − 434,164,620)	8,391.582 (7,381.141-9,367.389)				628,838,475 (551,834,407–700,881,341)	7,463.13 (6,575.679-8,321.797)	-0.381 (-0.406 - -0.355)
	DALYs	43,386,226 (31,083,937 − 58,355,210)	937.339 (669.132-1261.002)				70,156,962 (50,194,205 − 94,104,688)	832.179 (595.854-1115.244)	-0.383(-0.404 - -0.363)

#### Prevalence due to LBP in China and globally

Regarding the prevalence of LBP, China witnessed a rise in the count of LBP cases from 68,281,006 (95% CI: 59,158,022–77,853,897) in 1990 to 100,093,746 (95% CI: 87,128,173 − 113,014,316) in 2021, which corresponds to a cumulative augmentation of 46.591%. On a global scale, the prevalence of LBP escalated from 386,731,361 (95% CI: 341,581,662 − 434,164,620) in 1990 to 628,838,475 (95% CI: 551,834,407–700,881,341) in 2021, indicating a cumulative augmentation of 62.603%. In terms of the ASPR, globally, it declined from 8,391.582 (95% CI: 7,381.141-9,367.389) per 100,000 individuals in 1990 to 7,463.13 (95% CI: 6,575.679-8,321.797) per 100,000 individuals in 2021. Within China, the ASPR fell from 6,635.488 (95% CI: 5,770.679-7,472.799) per 100,000 individuals in 1990 to 5,342.1 (95% CI: 4,660.413-5,976.282) per 100,000 individuals in 2021. Furthermore, the AAPC in global prevalence dropped by 0.381% (95% CI: -0.406 to -0.355) from 1990 to 2021, and in China, it experienced a decrease of 0.682% (95% CI: -0.728 to -0.636)(Table 1).

#### DALYs due to LBP in China and globally

Globally, the DALYs for LBP escalated from 43,386,226 (95% CI: 31,083,937 − 58,355,210) in 1990 to 70,156,962 (95% CI: 50,194,205 − 94,104,688) in 2021, which corresponds to a 61.703% augmentation relative to the year 2021. Within China, the DALYs for LBP exhibited a 45.348% rise between 1990 and 2021. In terms of the ASDR, globally, it declined from 937.339 (95% CI: 669.132-1261.002) per 100,000 individuals in 1990 to 832.179 (95% CI: 595.854-1115.244) per 100,000 individuals in 2021. For China, the ASDR dropped from 749.026 (95% CI: 530.011-1013.842) per 100,000 individuals in 1990 to 603.033 (95% CI: 427.628–810.16) per 100,000 individuals in 2021. Furthermore, the average annual percentage change (AAPC) in global DALYs for LBP was a decrease of 0.383% (95% CI: -0.404 to -0.363) from 1990 to 2021, while in China, it was a more significant decrease of 0.682% (95% CI: -0.726 to -0.638)(Table 1).

### Joinpoint regression analysis of the burden of LBP in China and globally

**Fig. 1:**
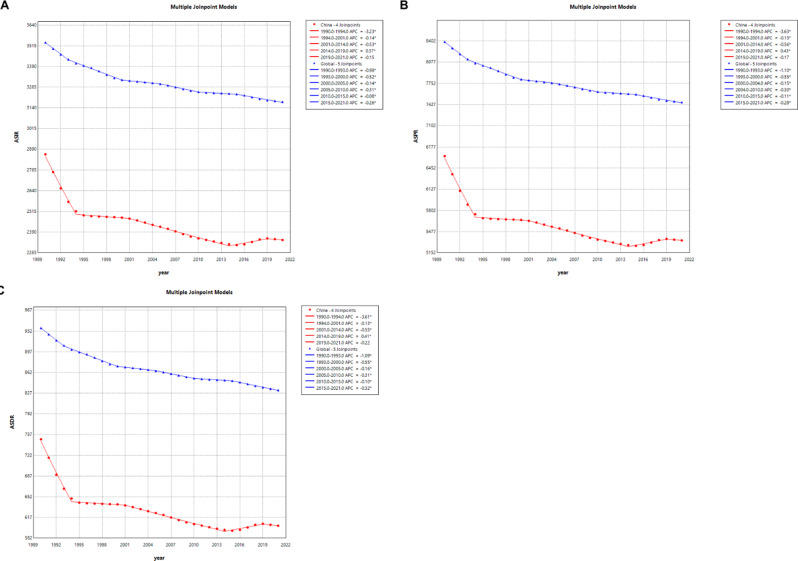
The APC in the ASIR, ASPR, and ASDR for LBP in China and worldwide from 1990 to 2021. (**A**) ASIR; (**B**) ASPR; (**C**) ASDR. (* indicates *p*-values < 0.05, denoting statistically significant results, the red curve represents China and the blue curve represents the worldwild)

The Joinpoint regression analysis highlights significant shifts in the trends of ASIR, ASPR, and ASDR for LBP in China and globally from 1990 to 2021, providing deeper insights into temporal and regional variations. (Fig. 1)

In China, the analysis revealed a significant decline in ASIR, ASPR, and ASDR between 1990 and 1994 (APC: -3.23, -3.63, and − 3.61, respectively; all *P* < 0.05), reflecting the initial impact of public health advancements and lifestyle changes. However, from 2014 to 2019, the data indicated a notable upward trend (APC: 0.37, 0.43, and 0.41, respectively; all *P* < 0.05), potentially linked to demographic shifts such as population aging and rising sedentary behaviors. This was followed by a marginal decline post-2019, reflecting variability in the burden of LBP.

In contrast, global trends displayed a consistent and stable decline across all metrics (ASIR, ASPR, and ASDR) from 1990 to 2021 (*P* < 0.05). The consistent global reduction highlights the sustained impact of improvements in healthcare, prevention strategies, and socioeconomic advancements.

By comparing these trends, the Joinpoint regression underscores China’s more pronounced variability and its distinct trajectory in contrast to global patterns. This analysis is crucial in identifying periods of significant change, enabling policymakers to design targeted interventions that account for these temporal and regional nuances.

### Gender disparities in the burden of LBP in different age groups in China and globally

**Fig. 2:**
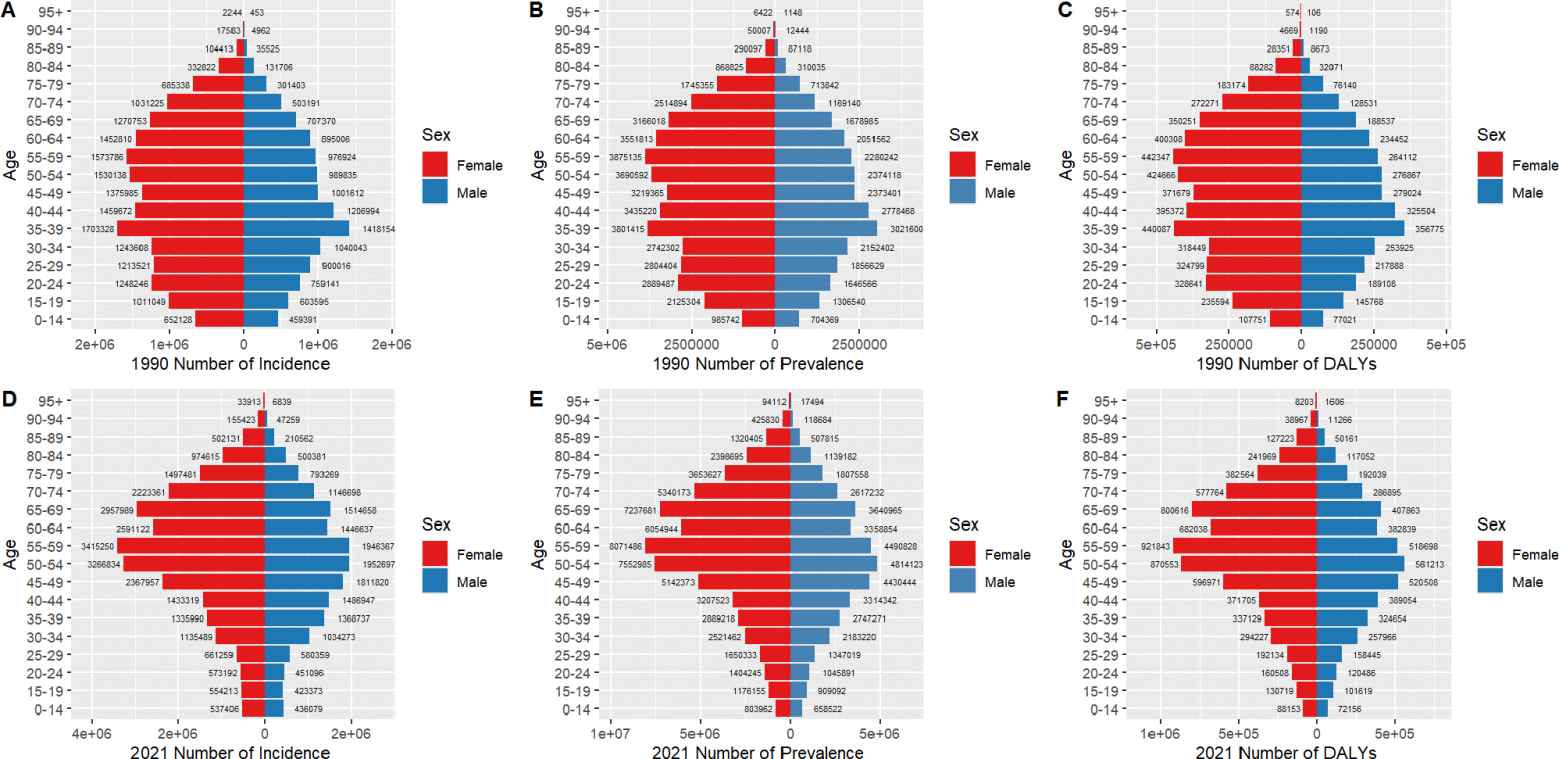
Comparison of the number of incidence, prevalence, and DALYs of LBP in males and females in different age groups in China in 1990 and 2021. (**A**) 1990 number of incidence; (**B**) 1990 number of prevalence; (**C**) 1990 number of DALYs; (**D**) 2021 number of incidence; (**E**) 2021 number of prevalence; (**F**) 2021 number of DALYs

**Fig. 3:**
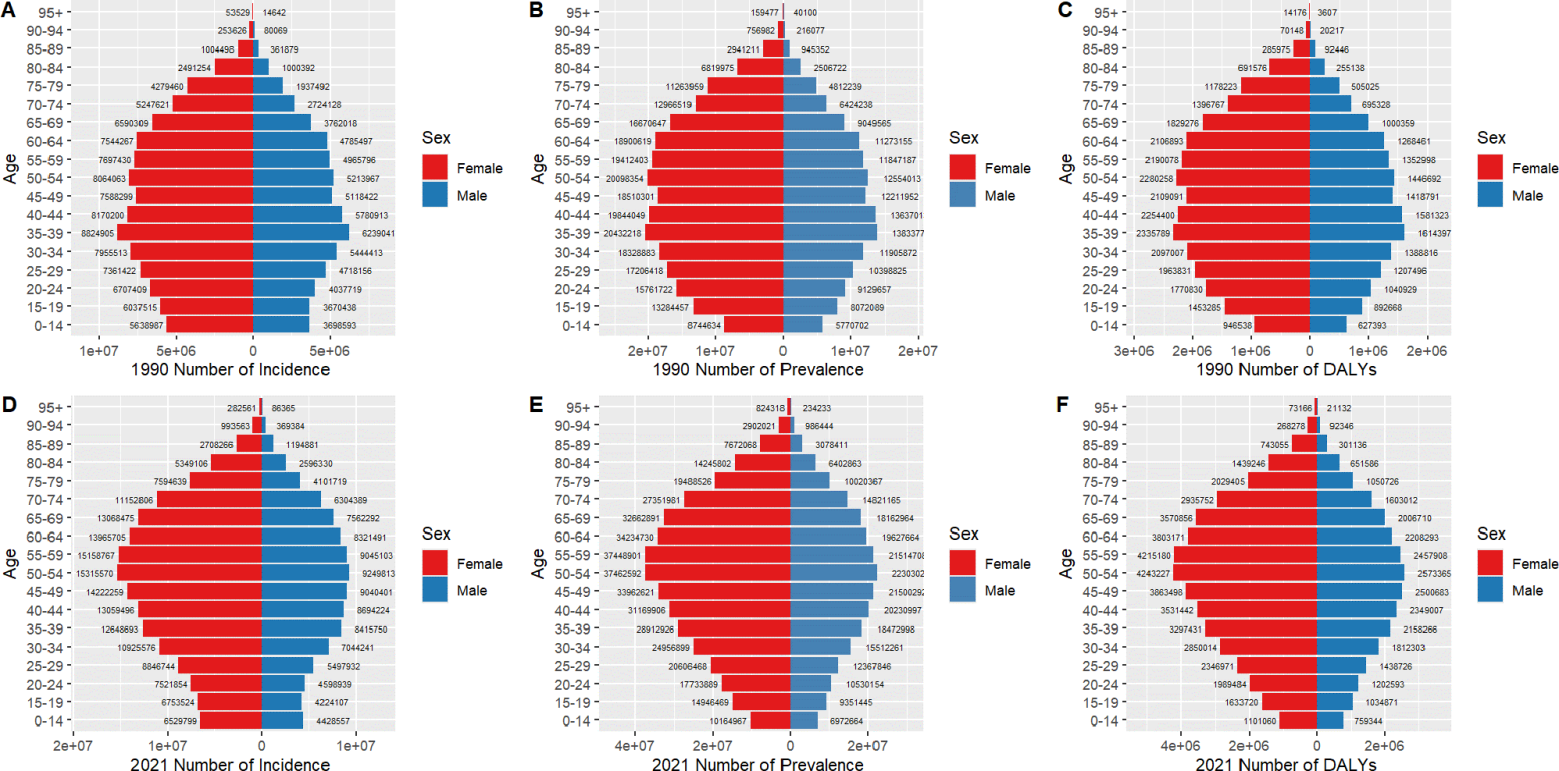
Comparison of the number of incidence, prevalence, and DALYs of LBP in males and females in different age groups in worldwide in 1990 and 2021. (**A**) 1990 number of incidence; (**B**) 1990 number of prevalence; (**C**) 1990 number of DALYs; (**D**) 2021 number of incidence; (**E**) 2021 number of prevalence; (**F**) 2021 number of DALYs

Figures 2 and 3 illustrate the incidence, prevalence, and DALYs of LBP across different age groups for males and females in China and globally, focusing on the years 1990 and 2021. The data reveal a consistently higher number of females affected by LBP across all age groups compared to males.

In China, the 1990 incidence data show that the number of LBP cases increased in both genders from the 0–14 age group, reaching a peak in the 35–39 age group. After age 39, the incidence declined for males, whereas females exhibited a secondary peak in the 55–59 age group. By 2021, the number of female LBP cases in China rose steadily from the 0–14 to the 55–59 age group, peaking at 55–59, with a secondary peak observed in the 65–69 age group. For males, the number of cases increased until the 50–54 age group, where it peaked, followed by a secondary peak in the 65–69 age group. Prevalence trends in 1990 for females showed a steady increase up to the 55–59 age group, peaking there, with a secondary peak observed at 35–39. Male prevalence trends in 1990 were similar to their incidence trends. By 2021, prevalence patterns for both genders closely mirrored the corresponding incidence trends. The DALY results also aligned with the prevalence data, showing peaks in females at 55–59 in both 1990 and 2021, while males peaked at 35–39 in 1990 and 50–54 in 2021.(Fig. 2).

Globally, incidence patterns in 1990 mirrored those observed in China, with peaks occurring in the 35–39 and 50–54 age groups for both sexes. In 2021, global incidence followed a similar trajectory, with peaks at 50–54 and declines thereafter. Global prevalence and DALY trends were consistent with the global incidence patterns (Fig. 3).

**Fig. 4:**
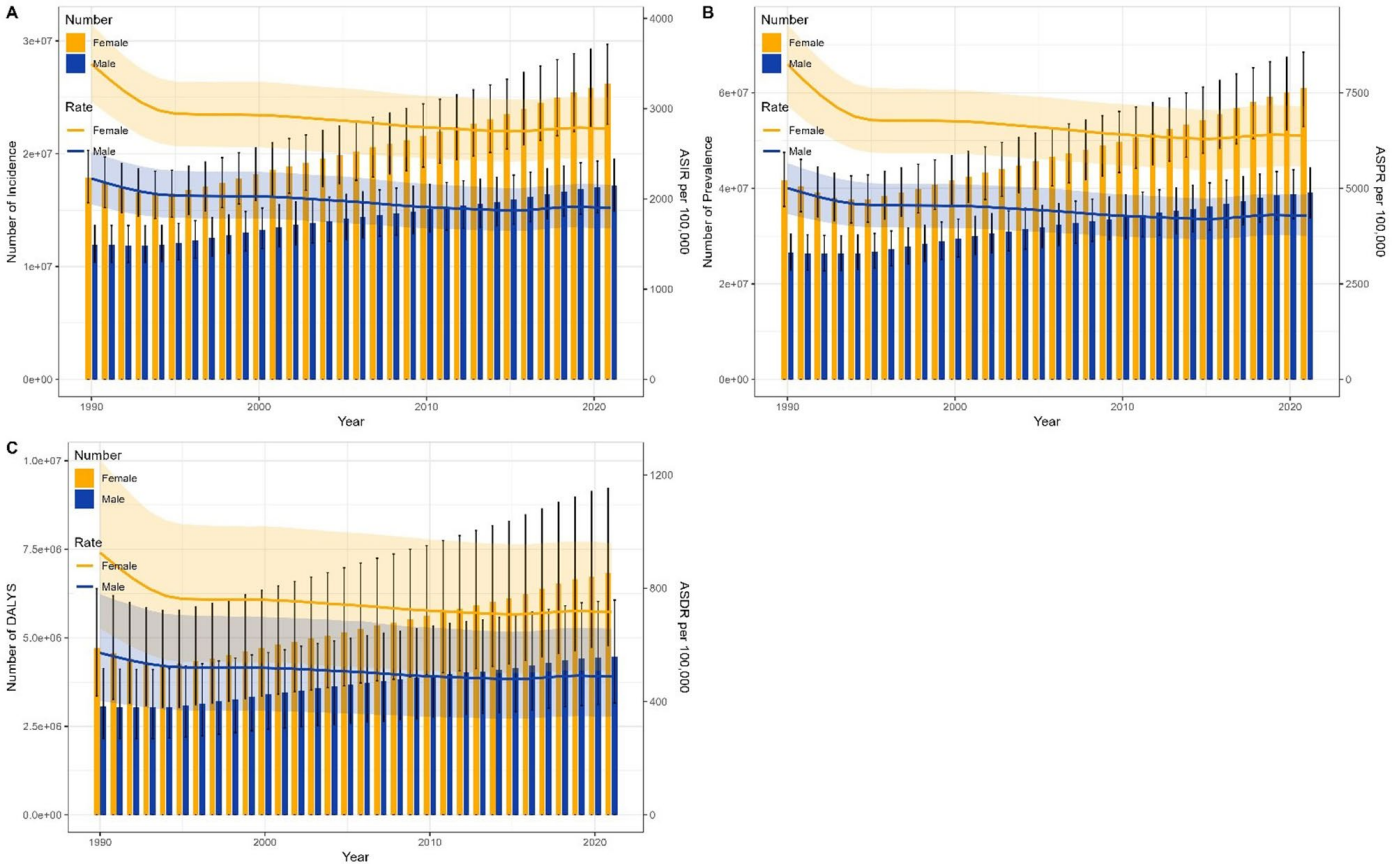
Comparison of all-age cases and age-standardized rates of incidence, prevalence, and DALYs among men and women in China from 1990 to 2021. (**A**) Number of Incidence and ASIR; (**B**) Number of Prevalence and ASPR; (**C**) Number of DALYs and ASDR. Bar charts display number data; lines depict age-standardized rates

**Fig. 5:**
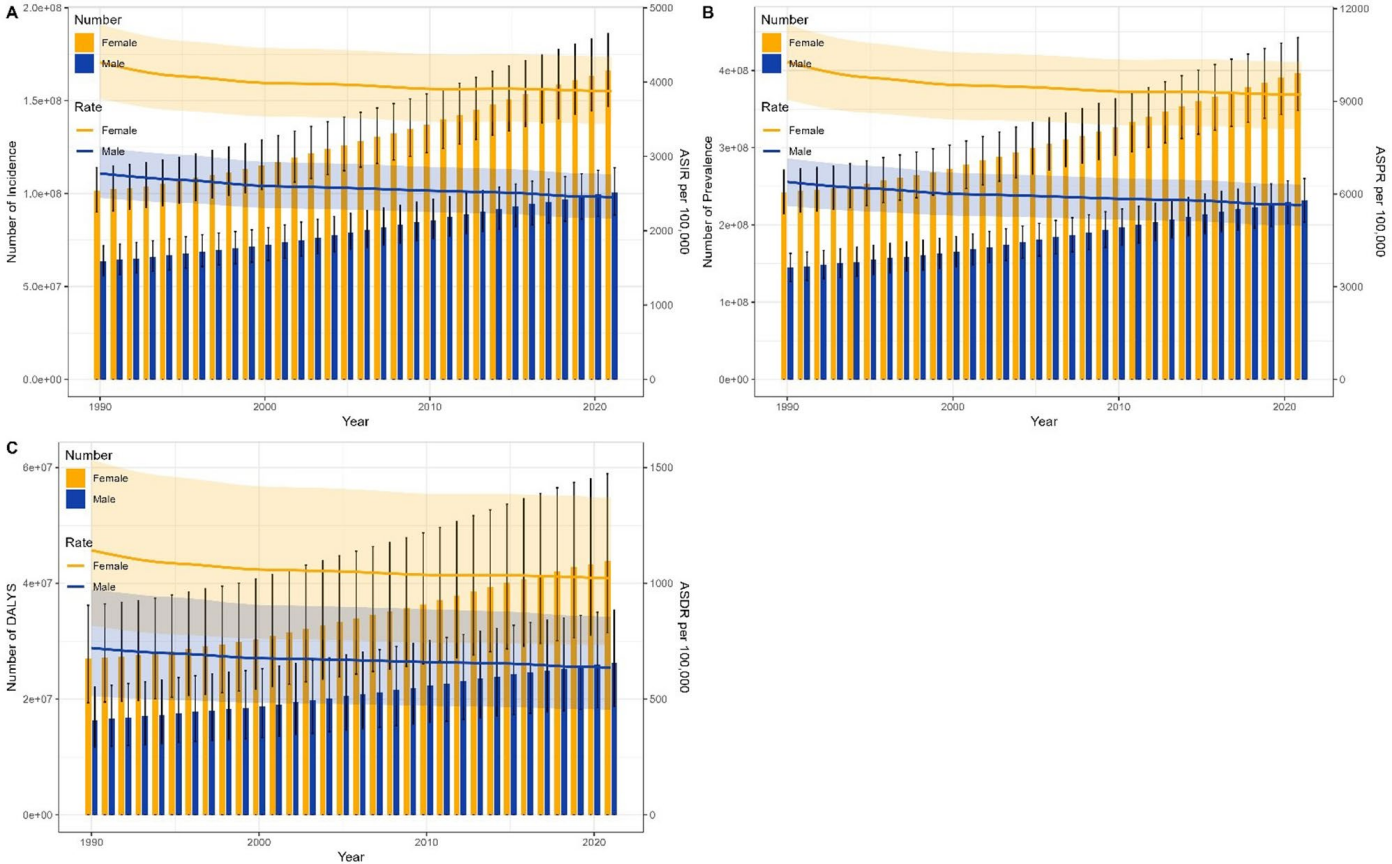
Comparison of all-age cases and age-standardized rates of incidence, prevalence, and DALYs among men and women in worldwide from 1990 to 2021. (**A**) Number of Incidence and ASIR; (**B**) Number of Prevalence and ASPR; (**C**) Number of DALYs and ASDR. Bar charts display number data; lines depict age-standardized rates

Figures 4 and 5 provided a comparative overview of the disease burden and LBP across genders for all age groups in China and globally, spanning from 1990 to 2021.

In China, the number of incidence, prevalence, and DALYs of LBP for both males and females have shown a consistent pattern since 1990. Initially, these numbers decreased from 1990 to 1994, reaching their lowest point in 1994. Subsequently, they gradually increased until 2021. Furthermore, during this period, the number of females consistently exceeded that of males. The ASIR, ASPR, and ASDR of LBP for both genders followed a similar trend as indicated by the Joinpoint regression analysis. Specifically, these rates exhibited a downward trend from 1990 to 2014, with the most pronounced decline occurring between 1990 and 1994. (Fig. 4)

Globally, the number of incidence, prevalence, and DALYs of LBP for both males and females have been on an upward trajectory from 1990 to 2021, with the number of females consistently exceeding that of males. In contrast, the ASIR, ASPR, and ASDR of LBP for both genders have shown a consistent downward trend over the same period, aligning with the findings of the Joinpoint regression analysis. (Fig. 5)

In summary, these findings underscore the increasing burden of LBP among middle-aged and older adults in both China and globally, with significant gender differences. These trends emphasize the need for age-targeted and gender-sensitive prevention and intervention strategies to mitigate the impact of LBP.

### Burden of LBP in different age groups in China and globally in 1990 and 2021

The analysis of LBP burden by age groups from 1990 to 2021 reveals distinct trends in China and globally.

In China, the age group with the highest LBP incidence shifted from 35 to 39 years in 1990 to 55–59 years in 2021, reflecting an aging population. This shift was mirrored in prevalence and DALYs, with both metrics peaking in the same age groups across these years. The CIR showed an age-related increase, peaking in the 80–84 years group in 1990 and the 85–89 years group in 2021. Similarly, the CPR peaked in the 85–89 years group for both years, while CDR peaked in the 75–79 years in 1990 and 85–89 years in 2021, highlighting the growing burden among elderly populations. (Supplementary Fig. 1)

Globally, a similar trend was observed. The age group with the highest LBP incidence shifted from 35 to 39 years in 1990 to 50–54 years in 2021. Prevalence and DALYs also mirrored these shifts. The global CIR trends paralleled those in China, peaking in the 75–79 years group in 1990 and the 80–84 years group in 2021. CPR globally peaked in the 80–84 years group for both years, while CDR demonstrated similar trends with the global CIR, reflecting a pronounced burden among older age groups worldwide. (Supplementary Fig. 2)

Overall, the findings emphasize the increasing impact of LBP in aging populations, with a more pronounced burden in elderly age groups both in China and globally. This underscores the need for age-targeted prevention and management strategies.

## Discussion

This study provides an in-depth examination of LBP over three decades (1990–2021) using data from the GBD study, focusing on China and global trends. The results revealed critical insights into the ASIR, ASPR, and ASDR, which consistently declined over the study period, with a more pronounced decrease in China compared to global averages. In terms of gender distribution, females displayed a heightened vulnerability to LBP. The incidence, prevalence, and DALYs of LBP were positively associated with patient age, with a notable increase in middle-aged individuals. Additionally, the CIR, CPR, and CDR for LBP were significantly higher among the elderly. Overall, while China’s LBP burden demonstrated a consistent decline, the gender and age patterns suggest a need for tailored public health interventions targeting middle-aged and elderly populations, as well as women who are disproportionately affected.

China’s reductions in ASIR, ASPR, and ASDR for LBP are particularly noteworthy, outpacing global averages. These improvements may be attributed to enhanced public health initiatives, economic development, and a growing emphasis on health and wellness in the population. For example, urbanization and increasing public awareness of the importance of physical activity and preventive care likely played pivotal roles [16]. Comparatively, global declines in LBP metrics were more modest, with significant regional disparities. Countries in South Asia and Europe experienced varying rates of decline, shaped by differences in healthcare infrastructure, lifestyle changes, and economic development [17, 18]. For instance, improvements in healthcare accessibility and policies targeting workplace ergonomics in high-income regions may explain their relatively lower burdens [19]. In contrast, low- and middle-SDI regions, including parts of Africa and Southeast Asia, face challenges such as limited healthcare access, lower awareness of LBP prevention, and economic constraints [20, 21]. These findings highlight the need for targeted strategies tailored to regional contexts, emphasizing the critical role of sociocultural and economic factors in shaping disease burden.

The study highlights significant gender and age-related disparities in the burden of LBP. Women consistently exhibited higher rates of incidence, prevalence, and DALYs than men, underscoring their heightened vulnerability to LBP. Biological factors, such as hormonal fluctuations, pregnancy, and the increased prevalence of osteoporosis and microfractures in elderly females, may partly explain this disparity [22]. Moreover, occupational patterns, such as prolonged sitting in sedentary jobs for women versus physical labor for men, also contribute to gender differences [23]. These findings align with previous studies demonstrating that women are more likely to report musculoskeletal pain and experience its disabling consequences. Age-related trends indicate that while crude rate peak in the 85–89 age group, the highest absolute burden of LBP occurs in middle-aged individuals (55–59 years in 2021). This shift toward middle age highlights the influence of postural imbalances, reduced flexibility, and occupational risks that accumulate over time [24]. The cyclical nature of pain, stemming from physical inactivity, musculoskeletal degeneration, and chronic conditions like intervertebral disc degeneration (IVDD), further exacerbates LBP prevalence and severity in aging populations [25, 26].

While the observed trends are promising, potential confounding factors must be considered. For example, smoking, obesity (BMI ≥ 28 kg/m²), and physical inactivity are well-established risk factors for LBP. Their interaction with age and gender-specific burdens warrants further investigation [27]. For instance, the higher rates of obesity in middle-aged populations may exacerbate the mechanical and systemic factors contributing to LBP [28]. Similarly, smoking-related effects on bone density and musculoskeletal health could disproportionately impact men engaged in physically demanding jobs [29].

Given the findings, actionable recommendations are essential to address LBP effectively. First, gender-sensitive approaches to prevention and treatment should be prioritized. For women, interventions focusing on osteoporosis prevention, ergonomic workplace adjustments, and awareness campaigns about the impact of hormonal changes on musculoskeletal health are critical [30–32]. Second, tailored public health strategies targeting middle-aged and elderly populations are necessary. These could include community-based physical activity programs, early screening for musculoskeletal conditions, and lifestyle modification initiatives to promote weight management and smoking cessation [33, 34]. Policymakers should also focus on integrating LBP management into primary healthcare systems, particularly in rural and underserved areas. Training healthcare providers in early detection and management of LBP, coupled with investments in rehabilitative services, can help mitigate the burden [35]. Furthermore, workplace policies aimed at improving ergonomics and reducing occupational risks, such as prolonged sitting or repetitive physical labor, are essential for preventing LBP in working-age populations [36].

While this study provides comprehensive insights, certain limitations must be acknowledged. The reliance on secondary data from the GBD study introduces potential biases stemming from variations in data quality across regions and time periods. For example, diagnostic techniques and data collection methods may have evolved over three decades, potentially affecting the accuracy of estimates. Additionally, the study’s macro-level analysis limits its applicability to specific regions or subpopulations within China or globally. Future research should focus on local-level data to inform region-specific interventions and explore the role of emerging factors, such as technological advancements and changing lifestyles, in shaping LBP trends.

## Conclusion

This study underscores the significant burden of LBP on individuals and healthcare systems in China and globally, despite observed declines in age-standardized metrics. The findings highlight the need for tailored, gender-sensitive, and age-specific interventions to address the complex interplay of biological, occupational, and lifestyle factors contributing to LBP. Policymakers, healthcare providers, and researchers must collaborate to implement targeted strategies, enhance public health education, and improve healthcare accessibility to reduce the global burden of LBP.

## Electronic supplementary material

Below is the link to the electronic supplementary material.

Supplementary Material 1

## Data Availability

The data sets that were produced and/or evaluated within the scope of this study can be accessed through the Global Burden of Disease (GBD) database repository, which is located at http://ghdx.healthdata.org/gbd-results-tool.

## Declarations

### Methods statement

All methods were executed in compliance with the applicable guidelines and rules.

## References

[1] Nicol V, Verdaguer C, Daste C, Bisseriex H, Lapeyre É, Lefèvre-Colau M-M, et al. Chronic low back Pain: a narrative review of recent International guidelines for diagnosis and conservative treatment. J Clin Med. 2023;12(4). 10.3390/jcm12041685. 10.3390/jcm12041685 PMC9964474 36836220

[2] GBD 2021 Low Back Pain Collaborators. Global, regional, and national burden of low back pain, 1990–2020, its attributable risk factors, and projections to 2050: a systematic analysis of the global burden of Disease Study 2021. Lancet Rheumatol. 2023;5(6):e316–29. 10.1016/S2665-9913(23)00098-X. 37273833 10.1016/S2665-9913(23)00098-X PMC10234592

[3] Zhou T, Salman D, McGregor AH. Recent clinical practice guidelines for the management of low back pain: a global comparison. BMC Musculoskelet Disord. 2024;25(1):344. 10.1186/s12891-024-07468-0. 38693474 10.1186/s12891-024-07468-0 PMC11061926

[4] Apeldoorn AT, Swart NM, Conijn D, Meerhoff GA, Ostelo RW. Management of low back pain and lumbosacral radicular syndrome: the Guideline of the Royal Dutch Society for Physical Therapy (KNGF). Eur J Phys Rehabil Med. 2024;60(2):292–318. 10.23736/S1973-9087.24.08352-7. 38407016 10.23736/S1973-9087.24.08352-7 PMC11112513

[5] Grooten WJA, Boström C, Dedering Å, Halvorsen M, Kuster RP, Nilsson-Wikmar L, et al. Summarizing the effects of different exercise types in chronic low back pain - a systematic review of systematic reviews. BMC Musculoskelet Disord. 2022;23(1):801. 10.1186/s12891-022-05722-x. 35996124 10.1186/s12891-022-05722-x PMC9394044

[6] GBD 2019 Diseases and Injuries Collaborators. Global burden of 369 diseases and injuries in 204 countries and territories, 1990–2019: a systematic analysis for the global burden of Disease Study 2019. Lancet. 2020;396(10258):1204–22. 10.1016/S0140-6736(20)30925-9. 33069326 10.1016/S0140-6736(20)30925-9 PMC7567026

[7] Zhang J, Tian Y, Li Y, Wang H, Yuan L, Zeng Y, et al. Time trends in the burden of low back pain and its associated risk factors in China from 1990 to 2019. J Orthop Translat. 2024;45:256–65. 10.1016/j.jot.2024.02.006. 38601199 10.1016/j.jot.2024.02.006 PMC11004195

[8] Wu Z, Huang G, Ai J, Liu Y, Pei B. The burden of low back pain in adolescents and young adults. J Back Musculoskelet Rehabil. 2024;37(4):955–66. 10.3233/BMR-230215. 38517768 10.3233/BMR-230215 PMC11321494

[9] Wang Y, Chen B, Liu X, Zeng H, Chen B, Wang Z, et al. Temporal trends in the burden of musculoskeletal diseases in China from 1990 to 2021 and predictions for 2021 to 2030. Bone. 2025;191:117332. 10.1016/j.bone.2024.117332. 39551255 10.1016/j.bone.2024.117332

[10] Zhang C, Zi S, Chen Q, Zhang S. The burden, trends, and projections of low back pain attributable to high body mass index globally: an analysis of the global burden of disease study from 1990 to 2021 and projections to 2050. Front Med (Lausanne). 2024;11:1469298. 10.3389/fmed.2024.1469298. 39507709 10.3389/fmed.2024.1469298 PMC11537905

[11] Yang W-L, Jiang W-C, Peng Y-H, Zhang X-J, Zhou R. Low back pain in China: Disease burden and bibliometric analysis. World J Orthop. 2024;15(12):1200–7. 10.5312/wjo.v15.i12.1200. 39744725 10.5312/wjo.v15.i12.1200 PMC11686520

[12] Wei J, Chen L, Huang S, Li Y, Zheng J, Cheng Z, et al. Time trends in the incidence of spinal Pain in China, 1990 to 2019 and its prediction to 2030: The Global Burden of Disease Study 2019. Pain Ther. 2022;11(4):1245–66. 10.1007/s40122-022-00422-9. 35969366 10.1007/s40122-022-00422-9 PMC9633916

[13] Zhu M, Zhang J, Liang D, Qiu J, Fu Y, Zeng Z, et al. Global and regional trends and projections of chronic pain from 1990 to 2035: analyses based on global burden of diseases study 2019. Br J Pain. 2024;20494637241310697. 10.1177/20494637241310697. 10.1177/20494637241310697 PMC11669129 39726775

[14] Diseases and Injuries Collaborators GBD. Global incidence, prevalence, years lived with disability (YLDs), disability-adjusted life-years (DALYs), and healthy life expectancy (HALE) for 371 diseases and injuries in 204 countries and territories and 811 subnational locations, 1990–2021: a systematic analysis for the global burden of Disease Study 2021. Lancet. 2024;403(10440):2133–61. 10.1016/S0140-6736(24)00757-8. 10.1016/S0140-6736(24)00757-8 PMC11122111 38642570

[15] Nijs J, Kosek E, Chiarotto A, Cook C, Danneels LA, Fernández-de-Las-Peñas C, et al. Nociceptive, neuropathic, or nociplastic low back pain? The low back pain phenotyping (BACPAP) consortium’s international and multidisciplinary consensus recommendations. Lancet Rheumatol. 2024;6(3):e178–88. 10.1016/S2665-9913(23)00324-7. 38310923 10.1016/S2665-9913(23)00324-7

[16] Jia N, Zhang M, Zhang H, Ling R, Liu Y, Li G et al. Prevalence and risk factors analysis for low back pain among occupational groups in key industries of China. BMC Public Health 2022, 22(1):1493. 10.1186/s12889-022-13730-8 10.1186/s12889-022-13730-8 PMC9354373 35931976

[17] Perera S, Ramani S, Joarder T, Shukla RS, Zaidi S, Wellappuli N, et al. Reorienting health systems towards primary Health Care in South Asia. Lancet Reg Health Southeast Asia. 2024;28:100466. 10.1016/j.lansea.2024.100466. 39301269 10.1016/j.lansea.2024.100466 PMC11410733

[18] Kumpunen S, Webb E, Permanand G, Zheleznyakov E, Edwards N, van Ginneken E, et al. Transformations in the landscape of primary health care during COVID-19: themes from the European region. Health Policy. 2022;126(5):391–7. 10.1016/j.healthpol.2021.08.002. 34489126 10.1016/j.healthpol.2021.08.002 PMC8364142

[19] Chen N, Fong DYT, Wong JYH. The global health and economic impact of low-back pain attributable to occupational ergonomic factors in the working-age population by age, sex, geography in 2019. Scand J Work Environ Health. 2023;49(7):487–95. 10.5271/sjweh.4116. 37634250 10.5271/sjweh.4116 PMC10838400

[20] Lim MY, Kamaruzaman HF, Wu O, Geue C. Health financing challenges in Southeast Asian countries for universal health coverage: a systematic review. Arch Public Health. 2023;81(1):148. 10.1186/s13690-023-01159-3. 37592326 10.1186/s13690-023-01159-3 PMC10433621

[21] Kodali PB. Achieving Universal Health Coverage in Low- and Middle-Income countries: challenges for Policy Post-pandemic and Beyond. Risk Manag Healthc Policy. 2023;16:607–21. 10.2147/RMHP.S366759. 37050920 10.2147/RMHP.S366759 PMC10084872

[22] Pedulla R, Glugosh J, Jeyaseelan N, Prevost B, Velez E, Winnitoy B, et al. Associations of gender Role and Pain in Musculoskeletal disorders: a mixed-methods systematic review. J Pain. 2024;25(12):104644. 10.1016/j.jpain.2024.104644. 39084479 10.1016/j.jpain.2024.104644

[23] Gomides LM, Abreu MNS, Assunção AÁ. Occupational inequalities and gender differences: work accidents, Brazil, 2019. Rev Saude Publica. 2024;58:13. 10.11606/s1518-8787.2024058005342. 38695442 10.11606/s1518-8787.2024058005342 PMC11037903

[24] Chen X, Chen K, Zou J, Editorial. Exercise for age-related musculoskeletal disorders. Front Public Health. 2023;11:1337093. 10.3389/fpubh.2023.1337093. 38222089 10.3389/fpubh.2023.1337093 PMC10787645

[25] Ma K, Chen S, Li Z, Deng X, Huang D, Xiong L, et al. Mechanisms of endogenous repair failure during intervertebral disc degeneration. Osteoarthritis Cartilage. 2019;27(1):41–8. 10.1016/j.joca.2018.08.021. 30243946 10.1016/j.joca.2018.08.021

[26] Blyth FM, Noguchi N. Chronic musculoskeletal pain and its impact on older people. Best Pract Res Clin Rheumatol. 2017;31(2):160–8. 10.1016/j.berh.2017.10.004. 29224694 10.1016/j.berh.2017.10.004

[27] Li Q, Peng L, Wang Y, Yang Y, Wang Z. Risk factors for low back pain in the Chinese population: a systematic review and meta-analysis. BMC Public Health. 2024;24(1):1181. 10.1186/s12889-024-18510-0. 38671417 10.1186/s12889-024-18510-0 PMC11055313

[28] Lee CA, Jang H-D, Moon JE, Han S. The relationship between change of Weight and Chronic Low Back Pain in Population over 50 years of age: a nationwide cross-sectional study. Int J Environ Res Public Health. 2021;18(8). 10.3390/ijerph18083969. 10.3390/ijerph18083969 PMC8069450 33918755

[29] Smith MA, Jackson A, Tobacco Use T, Cessation, Health M. Orthop Nurs. 2018;37(5):280–4. 10.1097/NOR.0000000000000479. 30247409 10.1097/NOR.0000000000000479

[30] Stevenson J. Prevention and treatment of osteoporosis in women. Post Reprod Health. 2023;29(1):11–4. 10.1177/20533691221139902. 36357006 10.1177/20533691221139902 PMC10009319

[31] Kodete CS, Thuraka B, Pasupuleti V, Malisetty S. Hormonal influences on skeletal muscle function in women across Life stages: a systematic review. Muscles. 2024;3(3):271–86. 10.3390/muscles3030024. 10.3390/muscles3030024 PMC12225299 40757596

[32] van Niekerk S-M, Louw QA, Hillier S. The effectiveness of a chair intervention in the workplace to reduce musculoskeletal symptoms. A systematic review. BMC Musculoskelet Disord. 2012;13:145. 10.1186/1471-2474-13-145. 22889123 10.1186/1471-2474-13-145 PMC3552974

[33] Montayre J, Neville S, Dunn I, Shrestha-Ranjit J, Wright-St Clair V. What makes community-based physical activity programs for culturally and linguistically diverse older adults effective? A systematic review. Australas J Ageing. 2020;39(4):331–40. 10.1111/ajag.12815. 32597566 10.1111/ajag.12815 PMC7818171

[34] Wilson K, Obesity. Lifestyle modification and behavior interventions. FP Essent. 2020;492:19–24. 32383844

[35] Duarte ST, Moniz A, Costa D, Donato H, Heleno B, Aguiar P, et al. Low back pain management in primary healthcare: findings from a scoping review on models of care. BMJ Open. 2024;14(5):e079276. 10.1136/bmjopen-2023-079276. 38754873 10.1136/bmjopen-2023-079276 PMC11097853

[36] Markova V, Markov M, Petrova Z, Filkova S. Assessing the impact of prolonged sitting and poor posture on Lower Back Pain: a Photogrammetric and Machine Learning Approach. Computers. 2024;13(9):231. 10.3390/computers13090231.

