# Supplementary material for: Analysis and comparison of the trends in burden of low back pain in China and worldwide from 1990 to 2021
Source: J Health Popul Nutr. 2025 Feb 13;44:39. doi: 10.1186/s41043-025-00768-8 (PMC11827349; doi:10.1186/s41043-025-00768-8)
Supplement: Supplementary file 1 — Supplementary Material 1 [file 41043_2025_768_MOESM1_ESM.docx]

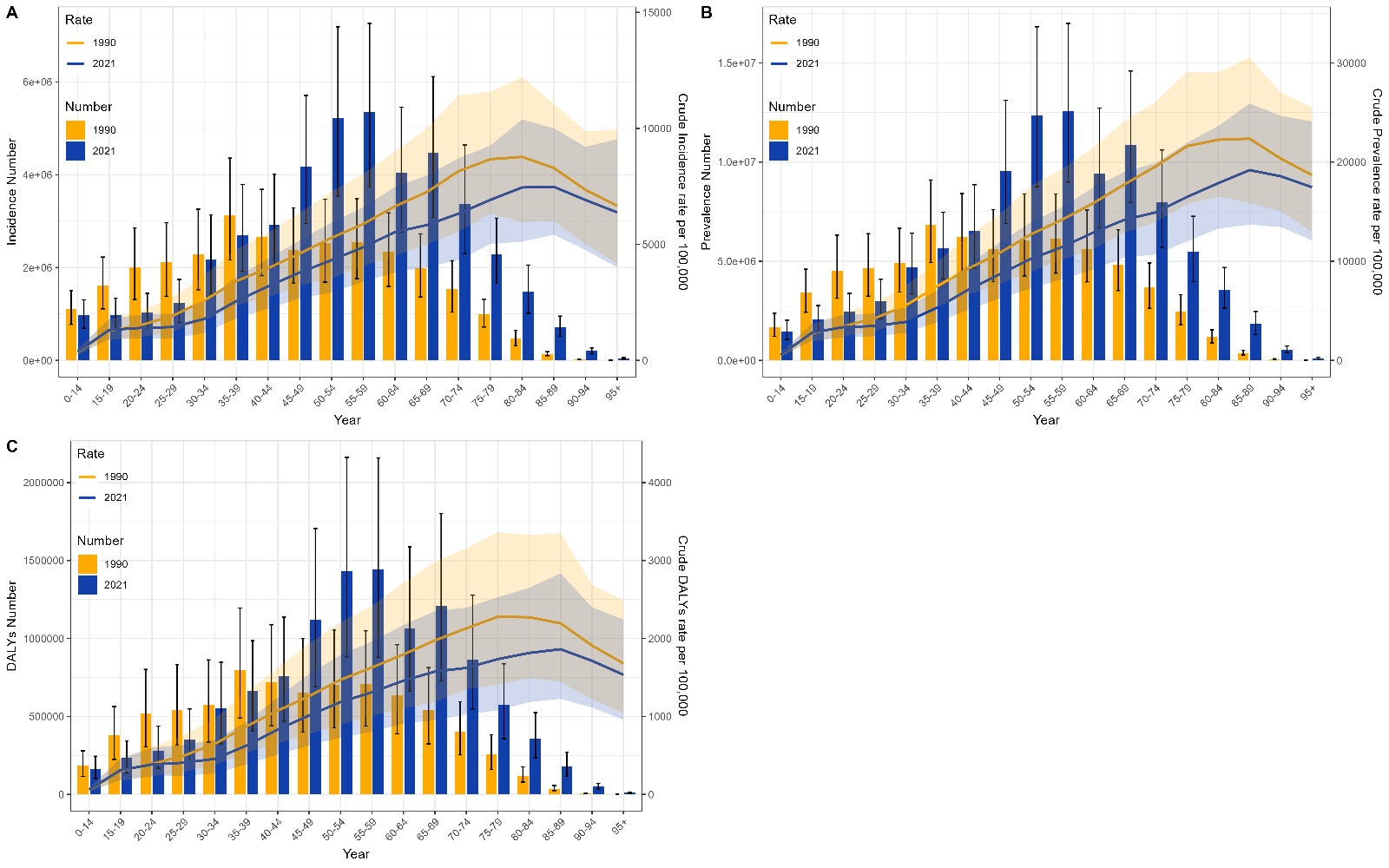


Supplementary Fig. 1 Comparison of the incidence, prevalence, and DALYs counts data, along with their crude rates, by age group from 1990 and 2021. (A) Incident cases and CIR of China; (B) Prevalent cases and CPR of China; (C) DALYs cases and CDR of China. Bar charts display count data; lines depict crude rates.


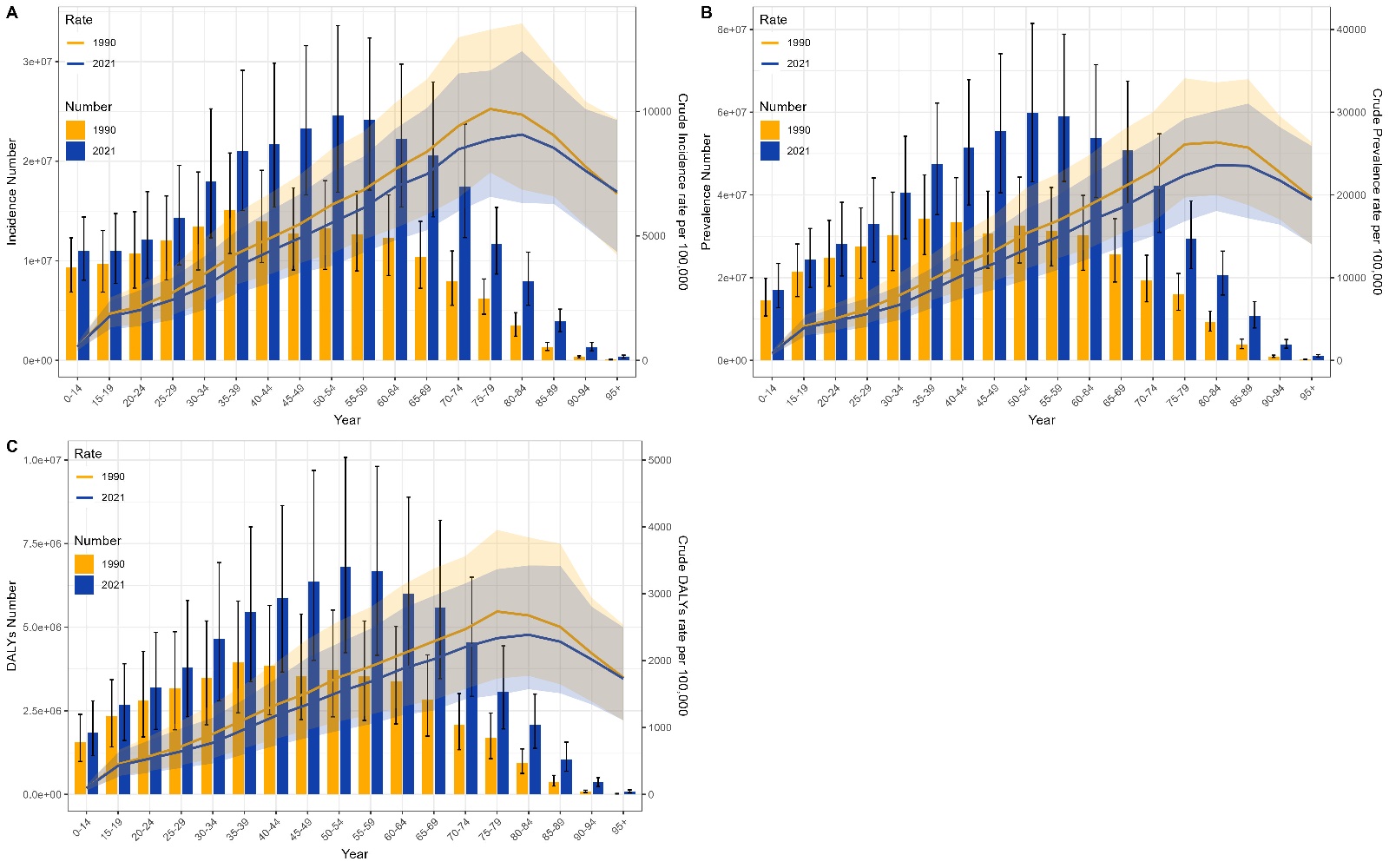


Supplementary Fig. 2 Comparison of the incidence, prevalence, and DALYs counts data, along with their crude rates, by age group from 1990 and 2021.(A)Global incident cases and CIR; (B) Global prevalent cases and CPR; (C) Global DALYs cases and CDR. Bar charts display count data; lines depict crude rates.

**Guidelines for Accurate and Transparent Health Estimates Reporting (GATHER)**

This study adhered to the **GATHER guidelines** to ensure transparency, reproducibility, and reliability in reporting health estimates. The following outlines how the principles were applied in this research:

1. **Data Inputs**:

Data were sourced from the publicly available **Global Burden of Disease (GBD) 2021 repository**.

Inputs included estimates for incidence, prevalence, years lived with disability (YLDs), and disability-adjusted life years (DALYs) derived from vital records, population surveys, health service utilization data, disease registries, and verbal autopsy reports.

The GBD platform (<http://ghdx.healthdata.org/gbd-results-tool>) was used for data extraction.

1. **Model Specifications**:

The study employed the **DisMod-MR** meta-regression model, which synthesizes data from multiple sources to estimate disease burden while addressing data gaps and inconsistencies.

Covariates such as socio-economic status, healthcare access, and environmental risk factors were incorporated to account for missing or incomplete data.

1. **Estimation Methods**:

Joinpoint regression analysis was used to determine the annual percentage change (APC) in LBP metrics (ASIR, ASPR, and ASDR) and to identify significant shifts in trends over time.

Logarithmic transformations were applied to linearize exponential trends and improve model fit.

1. **Uncertainty Quantification**:

Uncertainty intervals (95% CI) were calculated for all estimates to reflect data variability.

Sensitivity analyses were performed to validate model robustness and account for variations in data quality across regions.

1. **Validation**:

Comparisons with prior GBD studies and independent sources were conducted to confirm the consistency of results.

Internal cross-validation of the DisMod-MR model was performed to ensure reliability.

1. **Limitations**:

The study acknowledges residual confounding due to regional disparities in healthcare infrastructure and reporting quality.

Data gaps in low-income and rural regions, particularly in China, may influence the precision of estimates despite imputation efforts.

1. **Accessibility**:

All data and estimation tools used in this study are publicly accessible through the **GBD repository** (<http://ghdx.healthdata.org/gbd-results-tool>), ensuring full reproducibility.
